# Splicing time: how *WRKY55* isoforms regulate ABA signaling to balance growth and stress responses

**DOI:** 10.1093/plphys/kiag446

**Published:** 2026-06-30

**Authors:** Eva Maria Gómez-Álvarez

**Affiliations:** Assistant Features Editor, Plant Physiology, American Society of Plant Biologists; Instituto de Biología Molecular y Celular de Plantas (CSIC-Universitat Politècnica de València), Valencia 46022, Spain

The frequency and severity of environmental stresses, such as drought or salinity, have been increasing due to climate change, drastically affecting plant growth and crop yield (Sugumar et al. 2024). Plants undergo adjustment of several metabolic and signaling pathways that often involve transcriptional activation/repression, post-transcriptional modifications, and hormonal regulation. Abscisic acid (ABA), one of the most important and widely studied plant hormones, plays a vital role in plant responses to abiotic stresses (Mo et al. 2024; You et al. 2023; Tuteja 2007). Upon perceiving drought stress, plants accumulate ABA to trigger crucial survival mechanisms, such as stomatal closure during drought stress to prevent water loss. The ABA-mediated signal cascade is centered on transcription factors such as the basic leucine zipper (bZIP) ABSCISIC ACID-INSENSITIVE 5 (ABI5), which is a master regulator of ABA signaling (Skubacz et al. 2016; Balderas-Hernández et al. 2013).

Other players that are critical in this ABA regulatory network include the WRKY transcription factors, which modulate ABA signaling through binding to the conserved W-box cis-elements present in the promoters of the core ABA biosynthesis and signaling genes. WRKY transcription factors can activate and repress transcription of target genes, fine-tuning the response to stresses (Jiang et al. 2017). Besides the known ways plants turn down ABA signaling, alternative splicing has emerged as a key regulator, adding a further layer of post-transcriptional plasticity (Ma et al. 2026).

In a recent study published in *Plant Physiology*, researchers show how a single transcription factor gene, *WRKY55*, acts as a switch through alternative splicing (Quan et al. 2026). This mechanism coordinates both the activation and desensitization of ABA signaling to optimize plant stress resistance without being permanently detrimental for growth. ABI5, a central regulator in ABA-mediated growth inhibition, accumulates under stress to arrest development and to conserve resources. In the 2026 study, Quan et al discovered that WRKY55 is a direct upstream activator of ABI5 expression. Apparently, WRKY55 pre-mRNA is processed into 2 forms that encode 2 distinct protein isoforms with very different functions. The longer variant, WRKY55.1, holds all 3 exons and the functional DNA-binding WRKY domain. In response to short-term ABA treatment, *WRKY55.1* transcript and protein levels are rapidly increased, allowing the protein to bind directly to the *ABI5* promoter, driving stress-responsive gene expression. In contrast, the shorter variant *WRKY55.2* lacks the second exon, leading to the removal of the WRKY domain and making it unable to bind to the *ABI5* promoter. However, WRKY55.2 transcript levels only accumulate during prolonged ABA exposure. The accumulation of WRKY55.2 leads to the interaction of WRKY55.2 with WRKY55.1 in the nucleus, physically blocking WRKY55.1 from binding to the *ABI5* promoter, attenuating ABI5 expression and initiating the desensitization process.

The authors further refined the regulation of WRKY55-ABI5 by involving another transcription factor WRKY46. Under normal conditions without stress, WRKY46 interacts with WRKY55.1 to suppress premature ABI5 expression. However, when ABA is induced by stresses and plants initially perceive ABA, WRKY46 expression is downregulated, liberating WRKY55.1 to trigger ABI5 transcription. Under a prolonged exposure to ABA, this loop is shut down by an increase in WRKY55.2 expression, which will block WRKY55.1 from binding to ABI5 promoter (Fig. 1).

**Figure 1:**
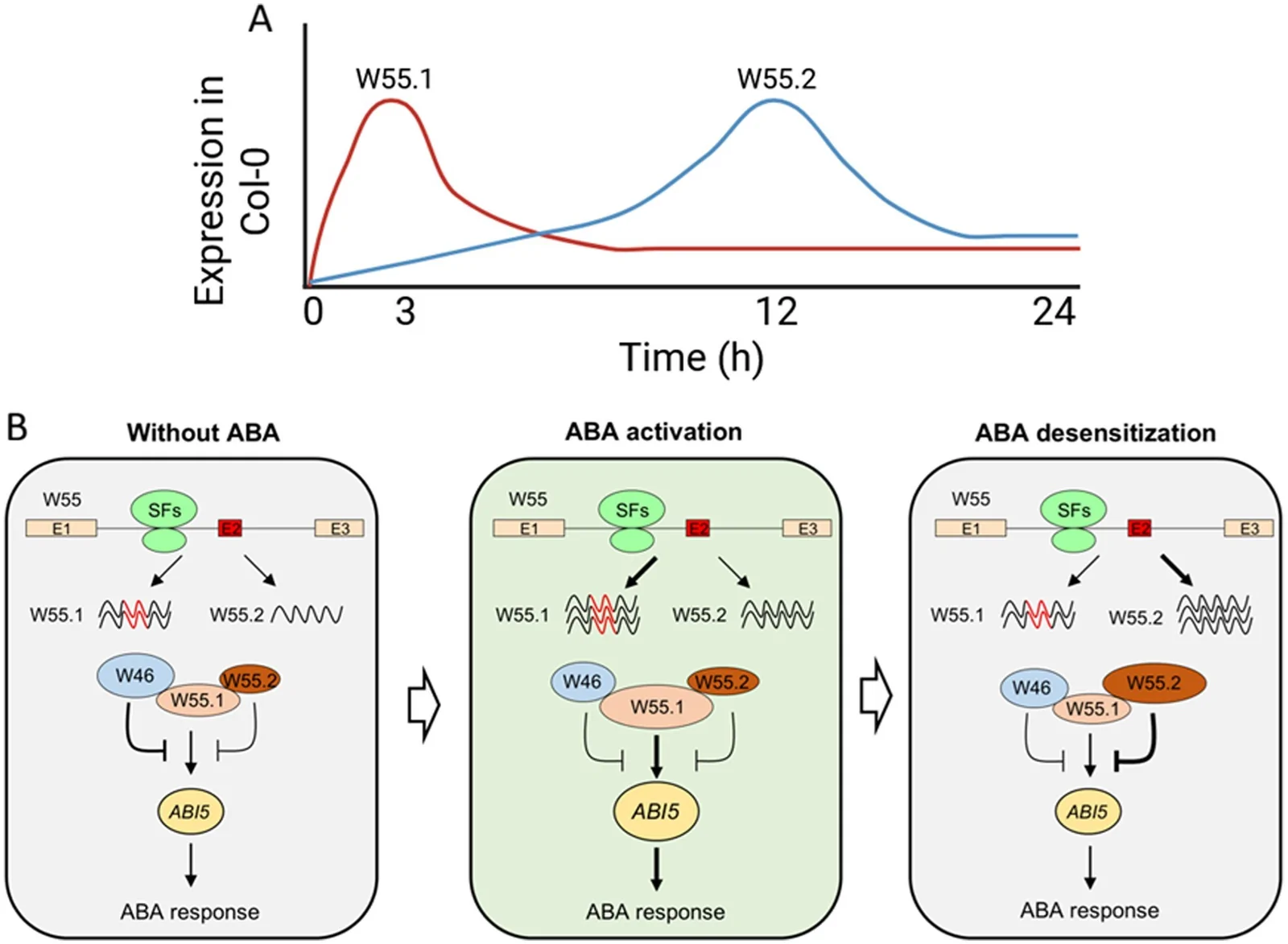
Alternative splicing of WRKY55 fine tunes ABI5 expression in response to stresses. A) Distinct expression kinetics of the W55.1 and W55.2 splice variants over time (interpretation of the data). B) Working model: how alternative splicing of WRKY55 modulates the activation and desensitization phases of the ABA response. Adapted from Quan et al. 2026. Figure performed with BioRender.

These findings show a novel mechanism of ABA signaling through alternative splicing. The findings also identified a crucial target for agricultural biotechnology. Elucidating the mechanisms that govern WRKY55.1/WRKY55.2 ratio could allow researchers to optimize plants stress responses without causing long-term growth inhibition.

## Data Availability

No new data were generated or analyzed in support of this research.

## References

[kiag446-B1] Balderas-Hernández VE, Alvarado-Rodríguez M, Fraire-Velázquez S. 2013. Conserved versatile master regulators in signalling pathways in response to stress in plants. AoB Plants. 5:plt033. 10.1093/aobpla/plt033. 24147216 PMC3800984

[kiag446-B2] Jiang J et al 2017. WRKY transcription factors in plant responses to stresses. J Integr Plant Biol. 59:86–101. 10.1111/jipb.12513. 27995748

[kiag446-B3] Ma S et al 2026. Regulation of alternative splicing in the ABA signaling pathway of plants. New Phytol. 250:115–124. 10.1111/nph.70846. 41404843

[kiag446-B4] Mo W et al 2024. Unveiling the crucial roles of abscisic acid in plant physiology: implications for enhancing stress tolerance and productivity. Front Plant Sci. 15:1437184. 10.3389/fpls.2024.1437184. 39640997 PMC11617201

[kiag446-B5] Quan S et al 2026. Alternative splicing of WRKY55 orchestrates ABA signaling to enhance plant stress resistance. Plant Physiol. 201:kiag422. 10.1093/plphys/kiag422. 42334883

[kiag446-B6] Skubacz A, Daszkowska-Golec A, Szarejko I. 2016. The role and regulation of ABI5 (ABA-insensitive 5) in plant development, abiotic stress responses and phytohormone crosstalk. Front Plant Sci. 7:1884. 10.3389/fpls.2016.01884. 28018412 PMC5159420

[kiag446-B7] Sugumar T, Shen G, Smith J, Zhang H. 2024. Creating climate-resilient crops by increasing drought, heat, and salt tolerance. Plants. 13:1238. 10.3390/plants13091238. 38732452 PMC11085490

[kiag446-B8] Tuteja N . 2007. Abscisic acid and abiotic stress signaling. Plant Signal Behav. 2:135–138. 10.4161/psb.2.3.4156. 19516981 PMC2634038

[kiag446-B9] You Z et al 2023. The CBL1/9-CIPK1 calcium sensor negatively regulates drought stress by phosphorylating the PYLs ABA receptor. Nat Com. 14:5886. 10.1038/s41467-023-41657-0. PMC10514306 37735173

